# Tumor microenvironment-mediated immune evasion in hepatocellular carcinoma

**DOI:** 10.3389/fimmu.2023.1133308

**Published:** 2023-02-10

**Authors:** Chen Chen, Zehua Wang, Yi Ding, Yanru Qin

**Affiliations:** Department of Oncology, The First Affiliated Hospital of Zhengzhou University, Zhengzhou, China

**Keywords:** hepatocellular carcinoma, immune evasion, tumor immune microenvironment, metabolism, gut microbiota, immunotherapy

## Abstract

Hepatocellular carcinoma (HCC) is the most common primary liver malignancy and is the third leading cause of tumor-related mortality worldwide. In recent years, the emergency of immune checkpoint inhibitor (ICI) has revolutionized the management of HCC. Especially, the combination of atezolizumab (anti-PD1) and bevacizumab (anti-VEGF) has been approved by the FDA as the first-line treatment for advanced HCC. Despite great breakthrough in systemic therapy, HCC continues to portend a poor prognosis owing to drug resistance and frequent recurrence. The tumor microenvironment (TME) of HCC is a complex and structured mixture characterized by abnormal angiogenesis, chronic inflammation, and dysregulated extracellular matrix (ECM) remodeling, collectively contributing to the immunosuppressive milieu that in turn prompts HCC proliferation, invasion, and metastasis. The tumor microenvironment coexists and interacts with various immune cells to maintain the development of HCC. It is widely accepted that a dysfunctional tumor-immune ecosystem can lead to the failure of immune surveillance. The immunosuppressive TME is an external cause for immune evasion in HCC consisting of 1) immunosuppressive cells; 2) co-inhibitory signals; 3) soluble cytokines and signaling cascades; 4) metabolically hostile tumor microenvironment; 5) the gut microbiota that affects the immune microenvironment. Importantly, the effectiveness of immunotherapy largely depends on the tumor immune microenvironment (TIME). Also, the gut microbiota and metabolism profoundly affect the immune microenvironment. Understanding how TME affects HCC development and progression will contribute to better preventing HCC-specific immune evasion and overcoming resistance to already developed therapies. In this review, we mainly introduce immune evasion of HCC underlying the role of immune microenvironment, describe the dynamic interaction of immune microenvironment with dysfunctional metabolism and the gut microbiome, and propose therapeutic strategies to manipulate the TME in favor of more effective immunotherapy.

## 1 Introduction

Hepatocellular carcinoma (HCC) is the most common primary liver malignancy and is the third leading cause of cancer-related mortality worldwide in 2020 (1). HCC frequently develops on a background of cirrhosis caused by multiple risk factors, including chronic viral infection of hepatitis B virus (HBV) or hepatitis C virus (HCV), alcohol abuse, aflatoxin exposure, non-alcoholic steatohepatitis (NASH), and drug-related liver injury (2). Treatment recommendations differ in various stages of HCC. The choice between locoregional treatments mainly depends on the tumor burden, location, and liver function (3). Based on clinical practice guideline, surgical resection, radiofrequency ablation (RFA), transarterial chemobolization (TACE), and liver transplantation are effective for tumor confined to the liver, whereas systemic therapy targeting the TME is available for unresectable HCC (3, 4). Since the first tyrosine kinase inhibitor (TKI) sorafenib was proven to extend the survival in advanced HCC patients without compromising liver function in 2008 (5), multi-TKIs and vascular endothelial growth factor (VEGF) inhibitors have been integrated into standard systemic therapy for advanced HCC (6–9).

Cancer immunotherapies have greatly revolutionized the clinical management of HCC in recent years, particularly the application of immune checkpoint inhibitor (ICI). It has been proven that the combination of atezolizumab (anti-PD1) and bevacizumab (anti-VEGF) was superior to the first-line treatment sorafenib (10). However, HCC continues one of the worst prognoses due to drug resistance and frequent recurrence. A large percentage of HCC patients still do not benefit from these immunotherapies or undergo immune-related adverse events. A potential explanation is these immune-based approaches primarily aim to reactivate dysfunctional T cell but ignore the immunosuppressive contribution of the tumor microenvironment (TME).

**Figure 1:**
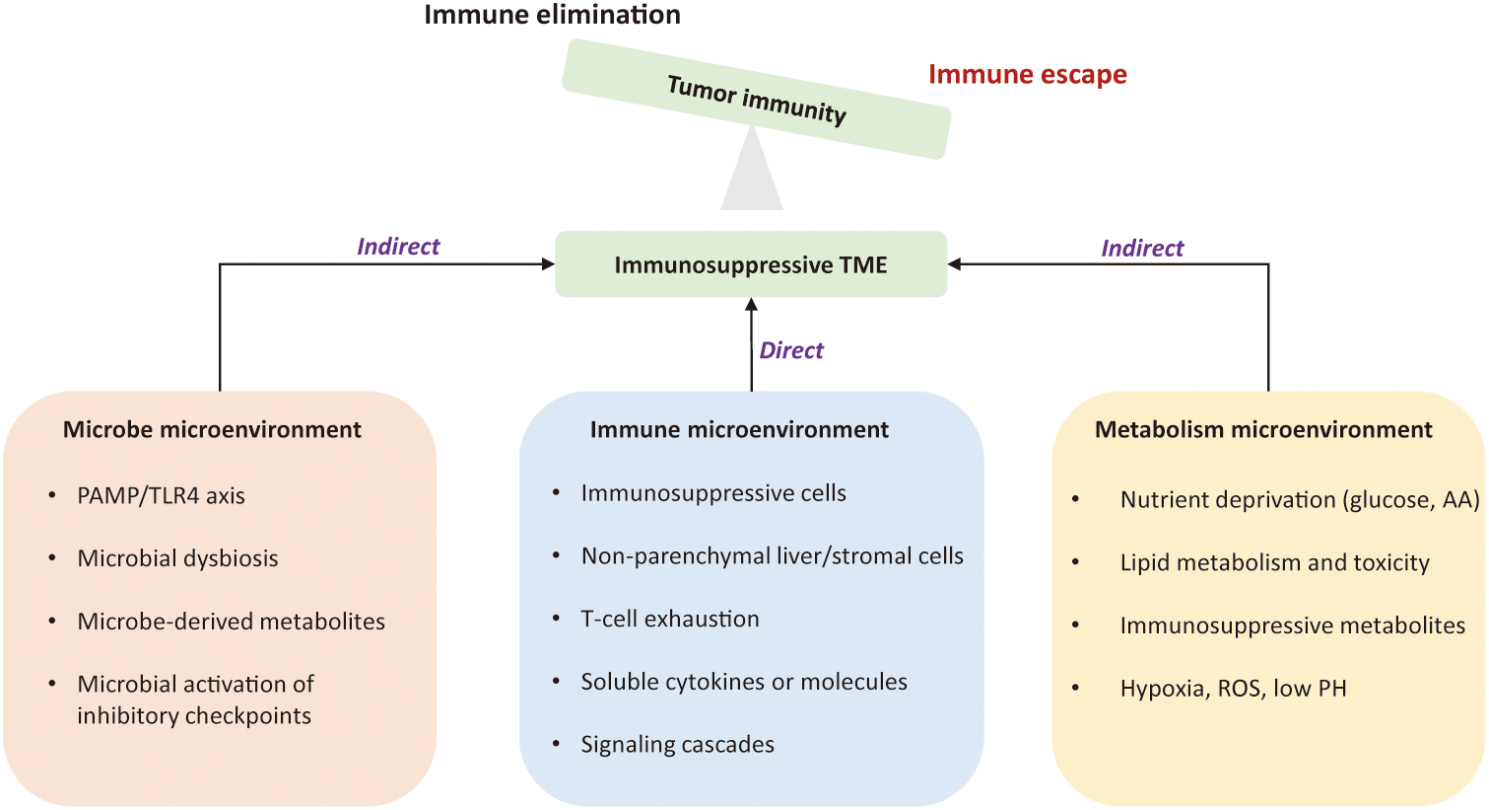
Mechanisms of immune evasion led by the tumor microenvironment in hepatocellular carcinoma. The immunosuppressive tumor microenvironment is an external driver of immune evasion in HCC. The suppressive immune microenvironment is led by intricate interactions among suppressive immune cells, stromal cells, immunoregulatory cytokines, and signaling cascades. Metabolic constraints and gut microbiota also contribute to the immunosuppression. The permissive microenvironment favors tumor cells to proliferate in un uncontrolled manner and is no longer confined by the host immunity. TME, tumor microenvironment; PAMP, pathogen-associated molecular patterns; TLR4, Toll-like receptor 4; AA, amino acid; ROS, reactive oxygen species.

The tumor microenvironment is a complex ecosystem that plays an indispensable role from cancer initiation to distant metastasis (11). It coexists and interacts with various immune cells and their products, referred to the tumor immune environment (TIME). Dysfunctional tumor-immunity cycle can lead to immune evasion by flawed antigen recognition or by immunosuppressive TME (12). Tumor intrinsic mechanism of immune evasion might be attributed to defects of antigen presentation, loss of MHC-I molecules, and epigenetic repression of tumor-associated antigens (TAA) (13). The immunosuppressive TME is an external driver of immune escape due to 1) the presence of immunosuppressive cells; 2) co-inhibitory signals on lymphocytes; 3) the existence of immunosuppressive soluble factors and signaling cascades; 4) metabolically hostile tumor microenvironment, imposing barriers to tumor-infiltrating immune cells; 5) the intra-tumoral microbes that alter the state of the immune microenvironment to prompt HCC progression (14–19). **Figure 1** depicts mechanisms of immune evasion mediated by tumor microenvironment in HCC.

The tumor immune microenvironment can determine whether immunotherapy will be successful. Importantly, gut microbiota and metabolism profoundly affect the immune microenvironment. Understanding their complicated interaction will contribute to better modulating HCC-specific immune response and overcoming resistance to already developed therapies. In this review, we provide an overview of immunosuppressive microenvironment in HCC, mainly introduce mechanisms of immune evasion underlying the role of immune microenvironment, gut microbial microenvironment, and metabolism microenvironment, and propose novel strategies to harness the TME to enhance HCC immunotherapy.

## 2 Immunoediting and immune evasion

Cancer immunoediting is a dynamic process that includes immune surveillance and tumor progression. It describes the relationship between tumor cells and immune system, proceeding through three phases: elimination, equilibrium, and escape (20). During the elimination phase, immune effector cells are able to recognize and eliminate tumor cells (20). In the equilibrium stage, tumor cells have escaped the elimination stage. But adaptive immunity still prevents the overall growth of the tumor, which keeps tumor cells in a state of functional dormancy (20, 21). In the escape stage, tumor cells continue to grow and proliferate in an uncontrolled manner and is no longer confined by the host immunity (20, 21). Tumor subclones that have acquired alterations could evade detection and destruction (20, 21).

**Figure 2:**
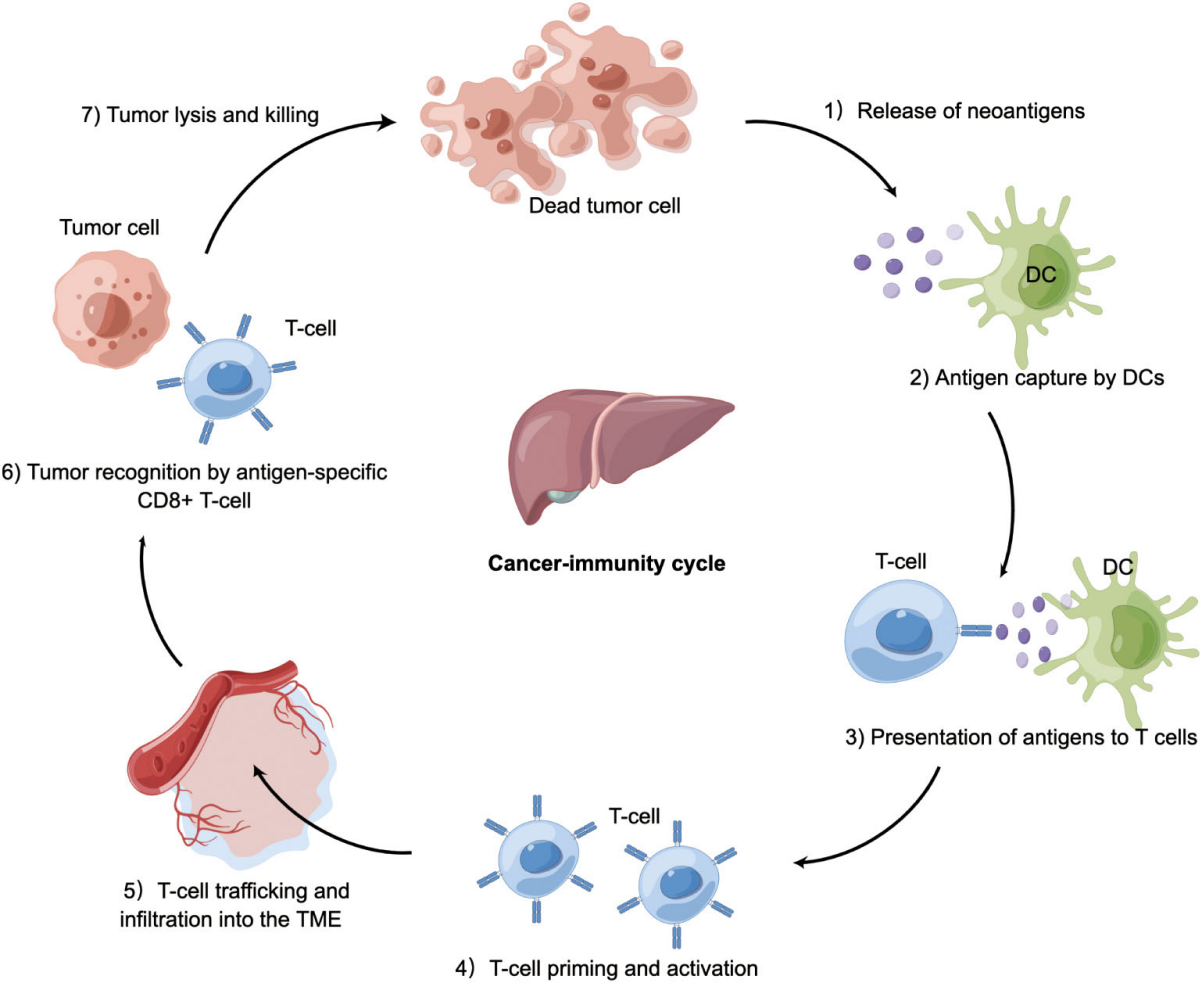
Cancer-immunity in HCC. Tumor cells release antigens into the tumor microenvironment due to necrosis or treatment. Dendritic cells capture cancer antigens and traffic to the lymphoid organs where they present antigens to T cells, followed by T-cell priming and activation. These activated T cells migrate and infiltrate into HCC tissue. CD8^+^ T cells recognize HCC cells *via* T cell receptor. The final step is T cell-mediated killing of tumor cells, allowing more cancer-specific antigens to release. Tumor can perturb the processes mentioned above to occur immune evasion. DC, dendritic cell; TME, tumor microenvironment; HCC, hepatocellular carcinoma.

The cancer-immunity cycle is a multistep process (**Figure 2**). The infinite proliferation and high tumor mutational burden of tumor cells firstly activate innate immune cells, such as natural killer (NK) cells, which target and lyse tumor cells to release tumor-associated antigens into the TME. These molecules are subsequently recognized by antigen-presenting cells (APCs), which travel to secondary lymphoid organs where adaptive immune responses are primed and activated (22). APCs present neoantigens to T cell receptor (TCR) of CD8^+^ cytotoxic T lymphocytes *via* the major histocompatibility complex (MHC) class I molecules. These activated T cells migrate and infiltrate into the HCC tissue. The final step is the T lymphocyte-mediated destruction of tumor cells, which in turn allows more tumor-associated antigens released into the TME (23, 24). Of note, the cancer-immunity cycle represents the adaptive aspect of immune surveillance phase (25–27). Tumors can perturb the processes mentioned above to evade immune surveillance by tumor-intrinsic mechanism (acquisition of genetic alterations) or tumor-extrinsic mechanism (generation of an immunosuppressive TME).

In acute infection, activated T cells can eliminate harmful pathogens. However, during the progression of HCC, these neoantigens are seldom eliminated, leading to the formation of chronic inflammatory stimulation that mediates the silence of the immune response and the loss of cytotoxic capacities of T cells. Previous studies have reviewed the escape of the tumor-intrinsic mechanism (28). The contributions of TME in this issue is usually be ignored. Therefore, the crosstalk among immune microenvironment, gut microbial microenvironment, and metabolic microenvironment is of great importance to HCC immune evasion.

## 3 Immune evasion mechanism in the immune microenvironment of HCC

**Figure 3:**
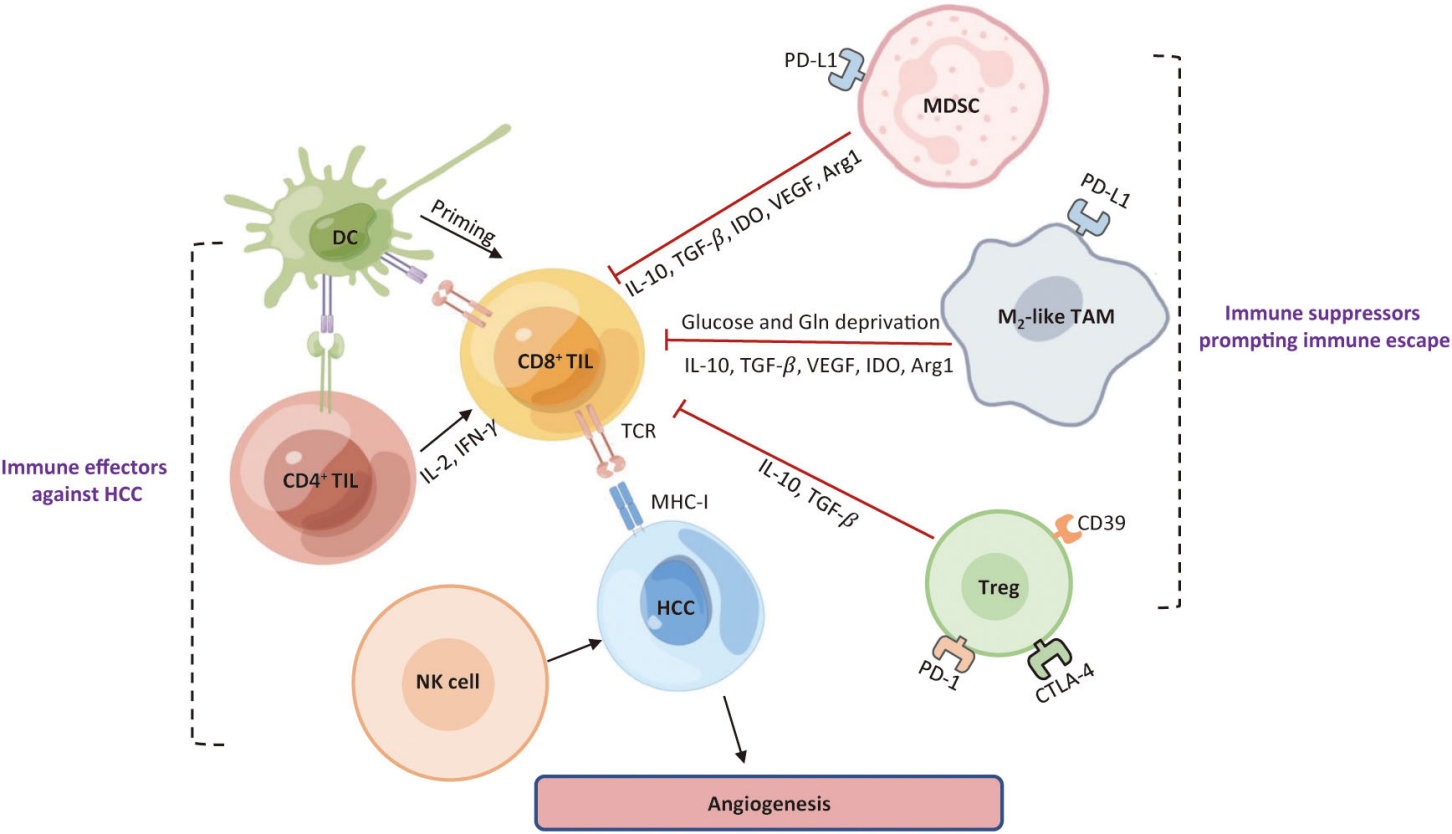
Roles of major immune cells in the HCC immune microenvironment. Immune cells existing in HCC can be roughly classified into one group that prompts an effective anti-tumor response, and the other group that limits immune response against HCC cells and contribute to an immunosuppressive TME. DC, dendritic cell; TIL, tumor-infiltrating lymphocytes; NK, natural killer; MDSC, myeloid-derived suppressor cell; Treg, regulatory T; TAM, tumor-associated macrophage; VEGF, vascular endothelial growth factor; TGF-β, transforming growth factor-*Β*; IDO, indoleamine 2, 3-dioxygenase; Arg1, arginase 1; Gln, glutamine; TCR, T cell receptor; MHC-I, major histocompatibility complex I; TME, tumor microenvironment; HCC, hepatocellular carcinoma.

Immune surveillance and evasion are respectively dictated by the opposing activities of effector immune cells and immunosuppressive cells in the TME (**Figure 3**). The hepatic TME is an intricate ecosystem that is comprised of tumor cells, immune cells, non-parenchymal liver cells, tumor-associated fibroblasts (29). Several lines of evidence suggest that the crosstalk between tumor cells and TIME components is a critical factor for the immune evasion of HCC and for the major cause of resistance to immunotherapies. The immunosuppressive milieu is consisted of immunosuppressive cells, non-parenchymal cells, T-cell exhaustion, soluble cytokines, and signaling cascades (30).

### 3.1 Immunosuppressive cells

Cytotoxic CD8^+^ T cells, CD4^+^ T cells, and NK cells work together to maintain immune surveillance, whereas abundant immune cells that resident in HCC contribute to immune evasion to prompt tumor progression, such as myeloid-derived suppressor cells (MDSC), regulatory T (Treg) cells, and tumor-associated macrophages (TAMs). Under physiological conditions, all populations participate in the manipulation of immune response, and thereby preserving homeostasis and self-tolerance (30, 31). However, both adaptive and innate immune response are blunted in HCC, as demonstrated by the TIME with dysfunctional TILs and NK cells (32–34).

MDSCs is a heterogenous group of immature myeloid cells that dampen CTL and NK cell effector functions, displaying a strong immunosuppressive activity in tumor-bearing hosts (35, 36). Several tumor-originated cytokines, such as IL-6, IL-1β, GM-CSF, G-CSF, VEGF, and MCP-1, have been reported to induce MDSC accumulation in preclinical models of HCC (37). An HCC-specific cell cycle-related kinase (CCRK) could upregulate IL-6 production *via* EZH2/NF-KB signaling, resulting in an extensive infiltration of polymorphonuclear MDSCs (38). Hypoxemia is a key regulatory factor that induces MDSCs accumulation *via* the chemokine C-C motif Ligand 26 (CCL26)/CX_3_CR1 pathway (39). Hypoxia-inducible factor 1α (HIF-1α) mediates ENTPD2 overexpression to convert ATP to 5’-AMP, which recruits a great quantity of MDSCs into the TME (40). Tumor-associated fibroblasts (CAFs) also facilitate the production of MDSCs by activating IL-6/STAT3 pathway (41). MDSCs accumulated in HCC could damage effector T cell function, reduce NK cell cytotoxicity, and expand immune checkpoint signaling, which blunt both innate and adaptive immune responses. The liver contains a large number of MDSCs that up-regulate the secretion of VEGF, TGF-β, and arginase, which inhibit T cell activation (42). MDSCs were found to deprive essential amino acids that are critical to T cell proliferation (43), and they release reactive oxygen and nitrogen species (iNOS or NOS2) that disrupt T cell receptor (TCR) signaling (44). Galectin-9 expressed on MDSCs binds to TIM-3 on T cells, which is associated with T cell apoptosis (45). Furthermore, a high infiltration of MDSCs in HCC is able to facilitate the conversion of naïve T cells into Treg cells (30). MDSCs also foster an immune escape status by reducing NK cell cytotoxicity. In senescent hepatocytes, MDSCs are recruited *via* the CCR2-CCL2 signaling, followed by differentiating into macrophages and blocking HCC initiation. However, once the tumor is initiated and developed, they would lose the ability of differentiation and cause inhibition of NK cell responses (46). Specifically, MDSCs can impair NK cell cytotoxicity by the NKp30 receptor and interact with Kupffer cells to enhance PD-L1 expression (47).

The physiological role of Treg cells is to inhibit excessive immune response to maintain homeostasis and autoimmune tolerance. However, hyperactive work of Treg cells in HCC supports tumor invasiveness, triggering a compromised T-cell immune response through several mechanisms (48–50). More CD4^+^ CD25^+^ Treg cells are enriched in the TME relative to that in in healthy individuals (51, 52). Treg cells are recruited by the chemokine receptor 6 (CCR6) and chemokine ligand 20 (CCL20) axis and activated by the binding of TCR with IL-10 and TGF-β signaling (53). Sorafenib, a multi-kinase inhibitor for HCC, has been proven to reduce hepatic Treg infiltration *via* suppressing TGF-β signaling (54). Long noncoding RNAs (lncRNA) are also involved in Treg cell differentiation (55). Specifically, the lncRNA-EGFR links an immunosuppressive state to HCC by augmenting activation of AP-1/NF-AT1 axis in Treg cells, thus prompting immune evasion (55). Overexpression of IL-35 has been shown to positively correlate with CD39^+^ FoxP3^+^ Treg cell infiltration, which may be another independent predictor for treatment efficacy among HCC patients (56). Mechanistically, CD4^+^ CD25^+^ FoxP3^+^ Treg cells could damage CD8^+^ T cell cytotoxicity by reducing the release of granzyme A, B, and perforin (57). Treg cells selectively inhibit some molecules that are essential in CD8^+^ T cell activation, such as TNF-α and IFN- γ (57, 58). Treg cell constitutively express CTLA-4 and secrete inhibitory molecules, such as IL-10 and TGF-β (59, 60).

As a significant component in the TME, TAM frequently portends a worse prognosis in HCC (61). TAMs arise from marrow-derived monocytes and obtain versatile immunosuppressive functions at each stage of differentiation. M_1_ and M_2_ are two polarizing phenotypes of TAMs with high plasticity in response to different stimuli. Substantial findings support that M1-polarized macrophages create pro-inflammatory cytokines and prevent malignancy development, whereas M_2_-polarized cells are able to produce tumor growth factor (IL-6), angiogenic molecules (VEGF), and immunosuppressive factors (Arg1, IL-10, TGF-β, and IDO) (62). Several HCC-originated cytokines, including IL-4, IL-13, CSF-1, CCL2, CXCL12, and CTG, promote CCR2^+^ inflammatory monocytes differentiation into TAMs in the TME (63–65). Moreover, TME-derived TGF-β facilitates TIM-3 expression on TAMs, fostering HCC development and immune tolerance (66). Osteopontin (OPN) correlates with PD-L1 upregulation and prompts TAM chemotaxis through the CSF1-CSF1 pathway (67). Under persistent hypoxia, HIF-1α/IL-1β loop between tumor cells and TAMs fosters epithelial-mesenchymal transition (EMT) and immune evasion (68). TAMs also produce cytokines and chemokines to drive immune suppression in HCC. For example, TAMs-derived CCL17, CCL18, and CCL22 could attract Treg cell infiltration into the TME (69, 70). The interplay between MDSCs and TAMs downregulates the production of IL-6, IL-12, and MHC-II but upregulates IL-10 secretion. TAM-derived IL-10 damages downstream CD8^+^ T cell and NK cell cytotoxicity but increases CD4^+^ CD25^+^ FOXP3^+^ Treg cell frequency (71, 72). Activated TAMs in the peritumoral stroma of HCC secrete a set of pro-inflammatory cytokines, such as IL-6, IL-23, IL-β, and TNF-α. These cytokines trigger the expansion of T helper 17 (Th17) cells that overexpress PD-1, CTLA-4, and GITR to exert an immunosuppressive function (73). Overall, TAMs might be a promising target for future HCC treatment.

Less common immunosuppressive cell types in human HCC consist of B cell population expressing PD-1, Th17 cells, CD4^+^ T cells expressing CCR4 and CCR6, CD14^+^ DCs expressing CTLA-4 and PD-1, tumor-associated neutrophils, tumor-associated fibroblasts, and type-II T helper cells (Th2) (74–77). These cells cooperate in the formation of immunosuppressive milieu and their presence usually manifests a poor prognosis in HCC.

### 3.2 Non-parenchymal liver cells

Liver is an immune organ with a number of immunocompetent cells. Non-parenchymal resident cells, such as Kupffer cells, hepatic stellate cells (HSC), and liver sinusoidal endothelial cells (LSEC), cooperate in the maintenance of immune tolerance.

Kupffer cells are liver-resident macrophages that act as antigen-presenting cells (APC) to form the first line of defense against pathogens (78, 79). Kupffer cells can contribute to hepatocarcinogenesis and immune escape underlying several mechanisms: 1) secretion of immunosuppressive cytokines (IL-10) (80); 2) upregulation of inhibitory immune checkpoint ligand PD-1 (81); 3) downregulation of costimulatory molecules (CD80 and CD86) (42, 82); 4) production of Indoleamine 2-3 dioxygenase (IDO) (83); 5) recruitment of Treg cells and T helper 17 (TH17) cells (42, 81, 82). The interaction of PD-L1 expressed by Kupffer cells and PD-1 expressed by T cells leads to T-cell exhaustion in human HCC (84). HSCs can secrete hepatocyte growth factor (HGF) that enables MDSC and Treg cells to accumulate inside the liver (85). Also, HSCs express high levels of PD-L1 to induce T cell apoptosis (86). LSECs not only motivate Treg cell activation *via* TGF-β but also highly express PD-L1 (87). Tumor-associated fibroblasts (TAF) can trigger NK cell dysfunction by secreting prostaglandin E2 (PGE_2_) and IDO, and prompt MDSC production by releasing IL-16 and CXCL12 (41).

### 3.3 T-cell exhaustion

Immune checkpoints involve co-inhibitory molecules preventing T-cell overactivation. Liver tumor cells and stromal cells express corresponding ligands to evade anti-tumor immunity (88). Co-inhibitory checkpoints include programmed cell death-1 (PD-1), cytotoxic T lymphocyte protein 4 (CTLA-4), lymphocyte-activation gene 3 (LAG3), T-cell immunoglobulin and mucin-domain containing 3 (TIM3), and others (88), acting as pivotal regulators of T-cell exhaustion (30, 31, 89).

CTLA-4 is expressed by activated T cells and is constitutively present on Treg cells. It prevents T cell proliferation and induces Treg cell activity inside HCC tissues (75, 90). PD-1 is expressed by activated T cells, NK cells, Treg cells, MDSCs, monocytes, and DCs, while its ligand, PD-L1, is mainly expressed by tumor and stromal cells. The interaction of PD-1/PD-L1 is suppressive for antigen-specific T cell activation (91–93). In HCC, high infiltration of PD-1^+^ CD8^+^ T cells predicts a worse prognosis and a higher risk of recurrence (94). In turn, overexpression of PD-L1 in tumor cells prompts CD8^+^ T cell apoptosis (94). The immune microenvironment of HCC also involves the overexpression of PD-L1 and PD-L2 in Kupffer cells, LSECs, and leukocytes (95).

The immunosuppressive roles of LAG3 and TIM3 have recently been uncovered in HCC. LAG3 that binds MHC-II molecules with high affinity, is upregulated upon T cell activation and is a molecular signature of T cell exhaustion (96). LAG3 expression is significantly higher on CD4^+^ and CD8^+^ tumor-infiltrating lymphocytes (TILs) than in other immune constituents among HCC patients (97). Similarly, TIM3 is expressed on CD4^+^ and CD8^+^ TILs, TAMs, NK cells in human HCC models (98). TIM3 interacts with its ligand galectin-9 mediating T-cell dysfunction (99), whereas its expression on Treg cells leads to enhanced suppressive activity (100). Notably, TIM3 is highly expressed in less differentiated tumor cells (101), which predicts poor prognosis in HBV-associated HCC (102).

Overall, immune checkpoints are expressed on the surface of T cells in different phases. Tumor cells evade immune-mediated destruction not only by expressing ligands to activate these receptors but also favor a suppressive TME by recruiting non-neoplastic cells to express these ligands. Immune checkpoint inhibitors (ICIs) are monoclonal antibodies designed to specifically disrupt inhibitory ligand-receptor interaction, removing T-cell exhaustion and recovering immune elimination (103–105) (**Table 1**). LAG3, TIM3, and PD-1 may function synergistically to facilitate HCC immune evasion and develop drug-resistance to PD1 or PD-L1 blockades (66, 106). Preclinical data support LAG3 and TIM3 inhibitors in combination with PD1 or PD-L1 ICIs, though their clinical values still require further elucidation.

**Table 1:** Immune checkpoint inhibitors and their targets in HCC.

Target	ICI	Clinical trial	Tumor type	Phase	Status	Enrollment
PD-L1	Atezolizumab	NCT04803994	Intermediate-stage HCC	III	recruiting	434
	Durvalumab	NCT05301842	Locoregional HCC	III	recruiting	525
	Sintilimab	NCT04220944	Unresectable HCC	I	recruiting	45
PD-1	Nivolumab	CheckMate-040	Advanced HCC	I-II	active	659
	Pembrolizumab	Keynote-224	Advanced HCC	II	active	156
	Tislelizumab	NCT03412773	Unresectable HCC	III	active	674
CTLA-4	Ipilimumab	NCT03682276	HCC	I-II	recruiting	32
	Tremelimumab	NCT01008358	Advanced HCC	II	completed	20
TIM-3	Cobolimab	NCT03680508	Advanced HCC	II	recruiting	42
LAG3	Relatilimab	None	None	None	None	None

HCC, hepatocellular carcinoma; ICI, immune checkpoint inhibitor; PD-1, programmed death 1; PD-L1, programmed death 1-ligand; CTLA-4, cytotoxic T lymphocyte-associated protein 4; TIM-3, T cell immunoglobulin and mucin domain containing-3; LAG-3, lymphocyte-activation gene 3.

### 3.4 Soluble molecules

The local milieu of cytokines and soluble mediators partly dictate the immune microenvironment of HCC. Considering a more complex layer, effects of these pleiotropic molecules greatly differ in their target immune cell population, or in acute or chronic inflammatory milieu (107). Non-parenchymal cells and infiltrating immune cells could secrete several cytokines and concurrently keep sensitive to these cytokines (108, 109). Secretion of TGF-β, IL-10, and VEGF into the TME all contributes to immunosuppression (42).

A well-identified example is TGF-β that is abundant in the TME of HCC. It could be generated by tumor cells, TAMs, and Treg cells and downregulates anti-tumor immunity at varying levels. Explicitly, TGF-β drives the polarization of TAMs into pro-tumorigenic M_2_-phenotype (110); favors the differentiation of naïve CD4^+^ T cells into Treg cells (111); impairs effector CD8^+^ T cell and NK cell cytotoxicity (112, 113); inhibits DC cell activation (114); and exert inhibitory effects on B cells (115). High serum TGF-β might predict poor anti-cancer response to sorafenib and pembrolizumab in HCC patients (116, 117). Evidently, TGF-β plays multitude effects on immune and tumor cells, hindering the inflammatory reaction and supporting immune evasion in HCC.

IL-10, a tolerance-inducing molecule in the HCC TME, is produced by tumor cells, TAMs, Treg cells, and DCs (118). It dampens the recruitment of tumor-infiltrating T cells (119) and upregulates PD-L1 expression in monocytes (120). High circulating levels of IL-10 have been shown to induce decreased TIL activity (121) and increased MDSCs (122). Increased plasma level of IL-10 portends to a poor prognosis in HCC patients (49, 123).

VEGF, a well-known regulator driving tumor angiogenesis, is mainly secreted by both tumor cells and the surrounding stroma (124). In addition to prompt angiogenesis, VEGF attenuates anti-tumor response by negatively affecting antigen-presenting cells (APCs) and effector T cells while maintains immunotolerant TME by positively increasing MDSCs and Tregs recruitment (125). Also, VEGF increases PD-1 expression on T cells and PD-L1 expression on TAMs. Focal gains at chromosome 6p21 leads to overexpression of VEGFA and thereby foster an immunosuppressive TME (126, 127). Overall, these findings build the fundamental to test the efficacy of drugs that counteract the immunosuppressive actions of TGF-β, VEGF, or IL-10 in HCC.

### 3.5 Signaling cascades

Tumor-intrinsic signaling cascades also affect the composition and function of HCC immune infiltrates. In a mouse model of HCC, CTNNB1 mutation or activation of WNT-β-catenin pathway could downregulate CCL5 expression and dampen DC recruitment, leading to immune escape and resistance to anti-PD-1 therapy (128). The expression of NKG2D ligand on HCC cells is also downregulated by β-catenin signaling, which is detrimental to the MHC-dependent immune response responsible by NK cells (129). Loss of p53 function facilitates the recruitment of immunosuppressive cells, and hepatoma CDK20 activation prompts the recruitment of MDSCs (38). In addition, overexpression of MYC, accounting for around 50-70% HCC cases, has been associated with PD-L1 upregulation (130). Finally, chronic HBV infection also results in overexpression of PD-L1 on Kupffer cells, leukocytes, and LSECs, and thus enhancing inhibitory signals in HCC TME (95, 131).

## 4 Immune evasion mechanism in the gut microbial microenvironment of HCC

The microbes reside within the tumor cells and immune cells. Increasing evidence suggests a critical link between the microbiota and the immune system (132–134). Intra-tumoral microbes and their products, defined as the tumor microbe microenvironment, have the potential to affect the tumor immune microenvironment. Gut microbiota is termed as a collection of microorganisms that colonize the intestine (135). Of note, the gut microbiota could repress immunosurveillance and prompt hepatocarcinogenesis. Understanding how gut microbes affect hepatic immune escape creates therapeutic innovations to improve HCC immunotherapy. The negative roles of microbes on TIME are multifaceted: 1) microbial activation of TLR4; 2) microbial dysbiosis; 3) microbe-derived metabolites; 4) microbial stimulation of inhibitory checkpoints.

### 4.1 PAMP-TLR4 axis mediates immune evasion

Microbial adjuvanticity is explained as the immunomodulatory function of the pathogen-associated molecular patterns (PAMP), which could be sensed by pattern recognition receptors (PRR). The most well-elaborated subtype of PRR is Toll-like receptor (TLR) (136). Microbial activation of TLRs contributes to the formation of immunosuppressive TME. TLR4 is considered to be one of the most important receptors to prompt hepatocarcinogenesis, which is expressed by hepatocytes, Kupffer cells, HSCs, LESCs, DCs, NKs, B cells, and T cells (137). Overexpression of TLR4 has been identified in HCC tumor samples (138, 139). TLR4 primarily recognizes lipopolysaccharide (LPS) that is a constituent of the cell wall of Gram-negative bacteria. LPS-induced TLR4 signaling is associated with microvascular invasion, early recurrence, and shortened survival in HCC patients (140).

Microbes mediate immune escape of HCC through direct or indirect TLR4-dependent manners. Firstly, TLR4 affects the recruitment and differentiation of various tolerance-inducing cells. Bacterial LPS recognized by TLR4 could stimulate hepatocytes to express CXCL1 that is a chemokine recruiting CXCR2^+^ polymorphonuclear MDSCs (141). Similarly, *Fusobacterium* recognized by TLR4 regulates IL-6/STAT3/C-MYC signaling pathway, facilitating TAM polarization into M_2_ phenotype (142). The interaction of TLR4 with macrophages indirectly prompts the accumulation of Treg cells in hepatoma cell lines, along with the upregulation of IL-10 and CCL22 (138). Secondly, LPS-induced TLR4 directly activates JNK/MAPK signaling to enhance the invasive ability and EMT of HCC cells (143). EMT enables epithelial cells to obtain mesenchymal characters to favor the formation of an immunosuppressive TME *via* upregulating co-inhibitory checkpoints and inducing resistance to NK cell-mediated lysis (144–146). The association between EMT and immunosuppression has been widely reported in HCC (147). Thirdly, LPS-mediated TLR4-AKT pathway upregulates the expression of Sox2, a stemness marker gene, thereby increasing the number of cancer stem cells (CSCs) of HCC (148). It is well known that CSCs are involved in immune evasion through certain intrinsic and extrinsic mechanisms (149). There is a tight association between TLR4 expression and CSC characteristics, contributing to the failure of immune surveillance (150). Furthermore, TLR4 is a direct target of microRNA-122 (miR-122), a tumor suppressor that inhibits the expression and activities of cytokines, such as VEGF, IL-6, COX-2, prostaglandin E_2_, and MMP-9 (151). Downregulation of miR-122 is linked to immune escape of HCC by targeting TLR4, which is associated with PI3K/AKT/NF-KB signaling pathway (151). Additionally, LPS-activated STAT3 signaling upregulates VEGF production for HCC angiogenesis (152). As discussed previously, VEGF is a key negative regulator of anti-tumor immunity.

Overall, these findings suggest that microbial stimulation of TLR4 can change the TIME. Intriguingly, drugs targeting TLR4 might be adjuvants to immune checkpoint inhibitors. Besides interacting with TLR4, a specific gut microbe can exert immunomodulatory effect *via* many different PRR-mediated signaling pathways, while some of them await further exploration (153).

### 4.2 Microbial dysbiosis mediates immune evasion

Maintenance of a balanced microbiota composition is crucial to forming an ecological barrier to insults from the external stimuli. The gut microbiota and mucosal immunity interact with each other to maintain intestinal homeostasis. Once this balance is disrupted, microbial dysbiosis would provide survival advantages for pathogenic bacteria along with decreased number of beneficial ones (154). An imbalance in gut microbiota composition is detected in HCC, with a significant increase of *E. coli* and *Atopobium* cluster while a significant regression of *Lactobacillus* species, *Bifidobacterium* species, and *Enterococcus* species (154). A recent study pointed out that a high cholesterol diet could induce gut microbial dysbiosis (depleted *Bifidobacterium* and *Bacteroides*) while altered flora metabolites in HCC patients (155).

Dysbiosis-mediated immune escape refers to a variety of mechanisms. Firstly, microbial dysbiosis can affect the content of immunogenic substances participating in intestinal homeostasis maintenance. High levels of lipopolysaccharide (LPS) have been detected in both pre-clinical models and HCC patients (154, 156), which is likely attributed to the leaky gut and bacterial translocation (157). Accumulation of circulating LPS from *Bacteroides* can prompt immune tolerance and hepatocarcinogenesis (156, 158). Likewise, TLR2 agonist lipoteichoic acid (LTA) can act on HSC to prompt senescence-associated secretory phenotype and enhance hepatocyte proliferation (159, 160). Secondly, microbial dysbiosis may alter the intracellular tight junction, thereby enhancing the interaction of dangerous signals with immune cells and facilitating the chronic inflammation (161–164). Previous studies supported that HCC often occurs in the context of chronic inflammation (165–167). Explicitly, some microbiota can invade colonic epithelial cells and activate intrinsic signaling pathways, aggravating the host inflammatory responses and releasing more cytokines (168, 169). Dysbiosis-driven chronic inflammatory can trigger oxidative stress that can deplete sensitive microbes and leave resistant strains (170). More importantly, it can mediate immune evasion by prompting angiogenesis, disrupting adaptive immunity, and altering the expression of pathogen recognition receptors (such as TLRs) and downstream signaling (171, 172). Overall, changes in microbiome composition are associated with the leaky gut (160, 173), endotoxemia, and systemic inflammation (174–176), predisposing the affected individuals more sensitive to developing HCC.

### 4.3 Microbe-derived metabolites mediate immune evasion

Microbial metabolites could enter the blood circulation and their receptors spread over both tumor cells and tumor infiltrating lymphocytes. Gut microbe-mediated bile acid metabolism regulates immune escape *via* decreasing the recruitment of NK T cells. Secondary bile acids (SBA) are derived from primary bile acids, of which process is mediated by gut microbes (177). SBA could downregulate the secretion of chemokine CXCL16 that interacts with CXCR6 to recruit NK T cells. Therefore, a reduced number of NK T cells through SBA *via* downregulating CXCR6-CXCL16, is beneficial for immune escape and HCC progression. Conversely, antibiotics that eliminate gut microbes could revert the above effects (178).

Deoxycholic acid (DCA) belongs to a gut bacterial metabolite that can induce DNA damage. A research confirmed that dietary or genetic obesity could result in microbial dysbiosis, thereby leading to an increasing level of DCAs (179). DCA has been shown to induce hepatic stellate cell senescence, thereby provoking the secretion of multiple cytokines that prompt hepatocarcinogenesis in mice model exposed to chemical carcinogen (179). Therefore, decreasing DCA level or targeting gut microbiota can specifically prevents immune evasion and inhibits HCC progression. Some other microbial-derived metabolites, such as N-acetylmuramic acid and N-acetylglucosamine, also exert their immunosuppressive effects on the TME (180).

### 4.4 Microbial activation of inhibitory checkpoints mediates immune evasion

The interactions between microbes and immune checkpoints could protect tumors from immune attack. The well-known inhibitory checkpoints include PD-1, CTLA-4, TIM-3, LAG-3, TIGIT, CEACAM1. Fap2 protein of *Fusobacterium mucleatum* binds to inhibitory receptor TIGIT or CEACAM1, repressing the activity of NK cells and effector T cells (181–183). The *helicobacter pylori* HopQ outer membrane protein interacts with CEACAM1 to inhibit immune cell activities (184). In addition, CD47 expressed by tumor cells can recognize its ligand SIRPα expressed by DCs and macrophages. CD47-SIRPα interaction could repress antigen presentation activity and phagocytosis (185). However, *Bifidobacterium* can upregulate the production of IFN-I in DCs, enhancing antigen presentation and T cell activation. Emerging evidence indicates that intravenous injection of *Bifidobacterium* could improve the efficacy of CD47 blockade (186). Overall, microbial stimulation of inhibitory checkpoints could manipulate HCC immune escape, but connections between microbes and inhibitory checkpoint deserve more investigation.

## 5 Immune evasion mechanism in the metabolic microenvironment of HCC

**Figure 4:**
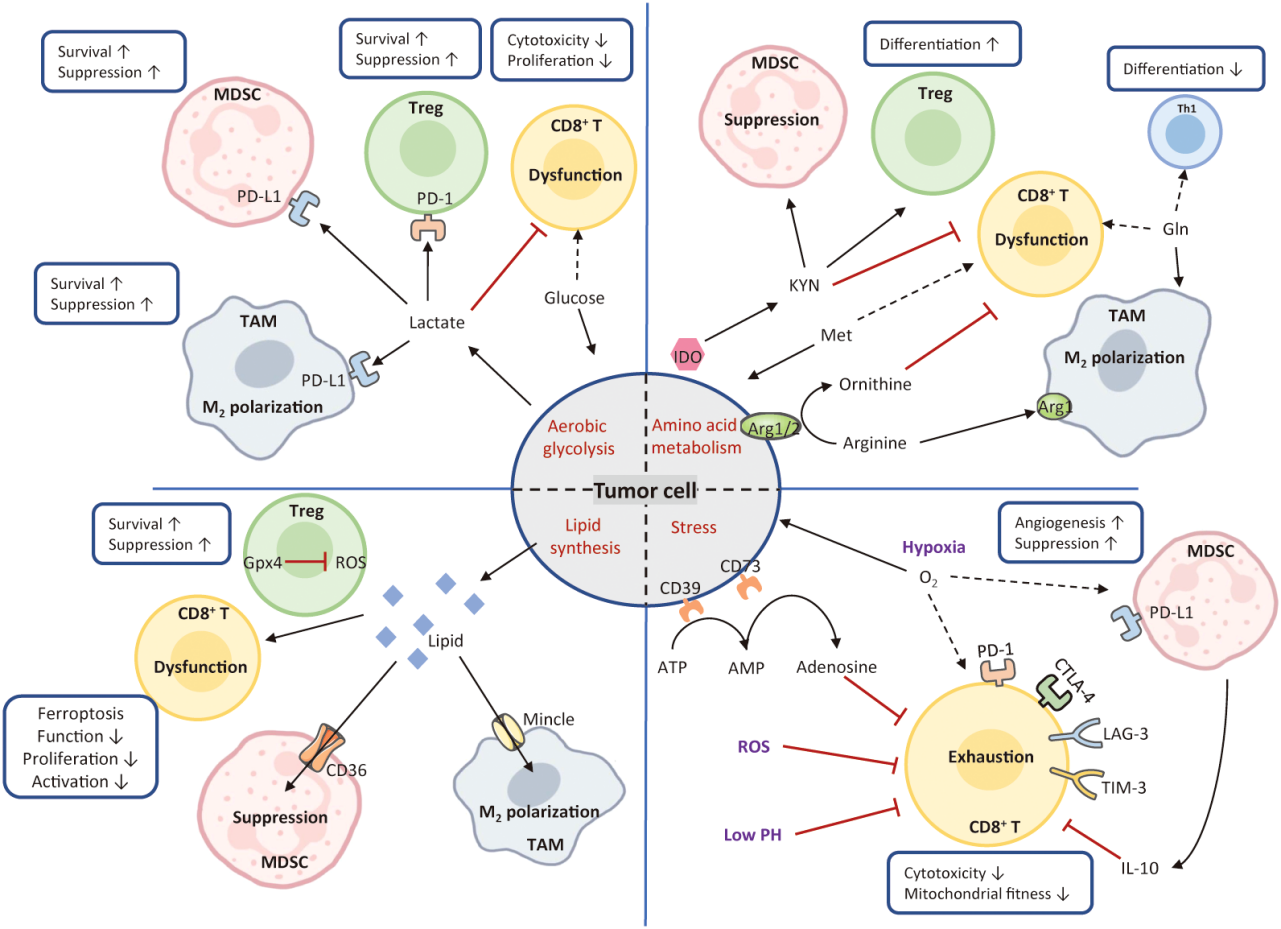
Mechanisms of metabolism-mediated immune escape. In the TME, hypermetabolic tumor cells interfere with immune cell function by depriving nutrients and produce various types of metabolic stress. Tumor cells utilize large amounts of glucose and amino acids to fuel their glycolysis and amino acid metabolism. These activities greatly limit nutrient availability to T cells, leading to the formation of immunosuppressive TME. Tumor cells also release excessive lipids into the TME, resulting in the enhanced lipid metabolism, high oxidative stress, and T-cell dysfunction. Conversely, Treg cells express high levels of glutathione peroxidase 4, avoiding ROS accumulation and the induction of ferroptosis. Cancer metabolism produces various metabolic stimuli, including hypoxia, low PH, and ROS, all of which impede CD8^+^ cytotoxicity and fitness. The solid black arrows present that the majority of nutrients are consumed by the cells, whereas the dashed black arrows indicate a paucity of molecule available to the cells. The red arrows represent inhibited metabolic pathways. MDSC, myeloid-derived suppressor cell; Treg, regulatory T; TAM, tumor-associated macrophage; Th1, T helper 1; IDO, indoleamine 2, 3-dioxygenase; Arg1, arginase; ROS, reactive oxygen species; KYN, kynurenine; Met, methionine; Gln, glutamine; Gpx4, glutathione peroxidase 4.

In response to external stress, such as nutrient competition, hypoxia, suppressive metabolites, tumor cells occur metabolic adaptions for survival from senescence and immune evasion. Understanding additional immunosuppressive mechanisms led by metabolic constraints would create a promising avenue to shift immune evasion to immune elimination (187). **Figure 4** introduces mechanisms of metabolism-mediated immune escape in HCC.

### 5.1 Glucose deprivation

Glucose is not only the most dependent nutrient for tumor cells, but also an essential energy source for immune cell activation, differentiation, and function (188, 189). Owing to the enhanced aerobic glycolysis, tumor cells consume a large amount glucose. This activity limits the glucose availability and results in lactate accumulation that acidifies the TME, severely impeding CD8^+^ T cell activation and function (190). Glucose restriction in TILs is found to reduce mTOR activity, glycolytic capacity, and IFN-*γ* production, and thereby immune cells gradually lose their effector functions (191, 192). By contrast, Treg cells can use lactate to fuel the tricarboxylic acid (TCA) cycle and support their survival under a low glucose environment (193). Moreover, M_2_-like TAMs and MDSCs can be highly glycolytic and use glucose to reinforce their survival and suppressive activity (194–196). In addition, lactate prompts TAM M_2_ polarization, MDSC differentiation, as well as PD-L1 expression in TAMs and MDSCs, contributing to immunosuppression (196–200).

### 5.2 Amino acid deprivation

Competition uptake for amino acids also contributes to immune escape (201, 202). For example, glutamine (Gln) deficiency in the TME inhibits effector T cell activation and reduces cytokine production (203). Also, Gln deprivation impairs Th1 cell differentiation while favoring Treg cell maintenance (204, 205). Intriguingly, TAM can enhance Gln synthetase to provide Gln and support TAMs in skewing towards the M_2_ phenotype even within a Gln-deficiency environment (206). Likewise, arginine (Arg) has been reported to be deprived in the TME. Arginase 1 (Arg1) or 2 convert arginine to ornithine that hampers CD8^+^ T cell activation and cytotoxicity (207). Conversely, Arg1 maintains the immunosuppressive property of MDSCs and facilitate repolarization of M_2_-like macrophages, consequently maintaining an immunosuppressive TME (208). In addition, tumor cells also outcompete T cells for methionine (Met). Met recycling pathway has been reported to drive T cell exhaustion in HCC (209).

### 5.3 Lipid metabolism and toxicity

Tumor cells display enhanced lipogenesis and produce a large amount of lipids in the TME. Immune cells uptake excessive lipids by CD36 or Mincle, leading to increased lipid metabolism and high oxidative stress. The direct consequences are T cell dysfunction and ferroptosis. However, Treg cells with high-level of glutathione peroxidase 4, prevents ROS accumulation and ferroptosis. Further, lipid-mediated endoplasmic reticulum stress prompt M_2_ differentiation and favors their suppressive function. Cholesterol homeostasis is disrupted due to the overexpression of acyl coenzyme A-cholesterol acyltransferase 1 (ACAT1), consequently accelerating the migration of HBV-related tumor cells while inhibiting the function of HCC-specific TILs (210, 211).

### 5.4 Metabolites

Metabolites existing in the HCC TME also hold immunomodulatory properties. Indoleamine-pyrrole 2,3-dioxygenase (IDO) is a heme-containing enzyme catalyzing the conversion of tryptophan to kynurenine. Its activation supports malignant cells to escape from immune clearance (30). Hyperactive IDO leads to the depletion of tryptophan from the TME contributing to T-cell anergy (212). Moreover, kynurenine accumulation upregulates PD-1 expression in effector T cells (213) and induce Treg cell production (214). IDO upregulation plays a role in drug-resistance to ICIs in patients with HCC. It has been confirmed that inhibiting IDO adds therapeutic benefits of ICI (215).

Adenosine is another immunosuppressive metabolite, concurrently impairing T cell functionality and prompting Treg cell proliferation (216, 217). Both tumor cells and MDSCs express ectonucleotidase CD39 and CD73 hydrolyzing ATP/ADP to adenosine (216). HCC patients with high levels of CD39 tend to have increased risk of recurrence and shortened overall survival (218). Overexpression of CD73 has been reported in human HCC cell lines, where it promotes HCC growth and metastasis (219).

### 5.5 Hypoxia

It is a common phenomenon that tumor cells consume excessive oxygen leading to an anoxia TME. HIF-1α is a major transcriptional factor that is upregulated in T-cell in response to hypoxia. First, hypoxia prompts the expression of inhibitory checkpoints, such as PD-1, LAG-3, TIM-3, and CTLA-4 (220). It also drives PD-L1 and IL-10 expression on MDSCs, which enhances their suppressive activity (221). Second, HIF-1α-induced EMT could create advantages for hepatoma cells to recruit IDO-overexpressing TAMs to repress T-cell response, and thereby facilitating immune escape *via* CCL20-dependent manner (147). Third, hypoxia-induced HIF-1α is detrimental to Treg cell differentiation and stability (222). Furthermore, HIF-1α binds to the promoter region of VEGF, followed by enhanced tumor angiogenesis (223). Hypoxia also aggravates the accumulation of lactate, which acidifies the TME and curtails effector immune cell function (224). Lactate contributes to the M_2_-like TAM polarization and maintains Treg cell function in a glucose-deficiency TME (197, 225). Under hypoxic condition, the COX-2/PGE_2_ axis stabilizes HIF-2α expression and activity to prompt HCC progression and develop drug-resistance to sorafenib (226). Overall, hypoxia can drive immunosuppression and exacerbate HCC immune escape.

## 6 Potential therapeutic strategies in the TME

Auspiciously, systemic therapies with molecular and immune therapies have remarkably revolutionized the management of HCC. Five single-agent molecular agents have been adopted by the US Food and Drug Administration (FDA) (3, 4, 227). In 2017 and 2018, two anti-PD-1 blockades, nivolumab and pembrolizumab, are approved as the second-line treatments for HCC (228). Notably, the superior results of atezolizumab plus bevacizumab versus sorafenib for advanced HCC heralded a new orientation of combination therapies (10). Currently, numerous clinical trials are in progress with ICIs, along with combined with anti-VEGF agents or tyrosine kinase inhibitor (TKIs). All approved drugs for HCC have been displayed in **Table 2**. A more refined understanding of the tumor microenvironment has led to great interests on ICIs. It is well evidenced that the immunosuppressive microenvironment in HCC triggers immune tolerance and escape by different mechanisms. Therefore, harnessing the TME by direct or indirect manners would provide new breakthroughs in HCC clinical treatment.

**Table 2:** FDA-approved drugs for hepatocellular carcinoma.

Drug	Classification	Target	Approval	Treatment	Clinical trial	Efficacy	Reference
Sorafenib	Multi-kinase inhibitor	BRAF, VEGFR, PDGFR, KIT	2007	First line	NCT00492752	OS: 10.7 VS 7.9 months (placebo)	(229, 230)
Regorafenib	Multi-kinase inhibitor	VEGFR, PDGFR, FGFR1, KIT, RET, BRAF	2017	Second line	NCT01774344	OS: 10.6 VS 7.8 months (placebo)	(7)
Nivolumab	ICI	PD-1	2017	Second line	CheckMate-040 CheckMate-459	ORR: 20% OS: 16.4 VS 14.7 months (sorafenib)	(231, 232)
Lenvatinib	Multi-kinase inhibitor	VEGFR, FGFR, PDGFR, RET, KIT	2018	First line	NCT01761266	OS: 13.6 VS 12.3 months (sorafenib)	(8)
Pembrolizumab	ICI	PD-1	2018	Second line	Keynote-224 Keynote-240	ORR: 17% OS: 13.9 VS 10.6 months (placebo)	(233, 234)
Cabozantinib	Multi-kinase inhibitor	VEGFR, MET, RET, KIT, AXL	2019	Second line	NCT01908426	OS: 10.2 VS 8 months (sorafenib)	(6)
Ramucircumab	Monoclonal antibody	VEGFR	2019	Second line	NCT02435433	OS: 8.5 VS 7.3 months (placebo)	(9)
Nivolumab + Ipilimumab	ICI plus ICI	PD-1 + CTL1-4	2020	Second line	CheckMate-040	ORR: 33%	(235)
Atezolizumab + Bevacizumab	ICI plus anti-VEGF	PD-L1 + VEGF	2020	First line	IMbravel	OS: 19.2 VS 13.4 months (sorafenib)	(10)

ICI, immune checkpoint inhibitor; OS, overall survival; ORR, overall response rate; VEGFR, vascular endothelial growth receptor; PDGFR, platelet-derived growth factor receptor; FGFR, fibroblast growth factor; PD-1, programmed cell death-1; PD-L1, programmed cell death ligand 1; CTLA-4, cytotoxic T lymphocyte-associated antigen-4.

### 6.1 Targeting the immune microenvironment

A promising approach is to deprive or neutralize cells with immunosuppressive functions. MDSCs have been considered as a potential target for resetting the immune tolerance status of HCC. Trabectedin not only targets malignant cells but also induces apoptosis or senescence of bone marrow cells (236). It has been reported to exert a strong cytotoxic effect on HCC cells (237). Another agent is estrogen that reportedly reduces IL-6 stimulation and inhibits STAT6 activation, leading to the disruption of bone marrow cells in HCC models (238, 239). The combination therapy of anti-PD1/PD-L1 and anti-MDSCs (CCRK inhibition, p38 MAPK inhibitor, and C5AR blockade) may exert a synergistical effect on eradicating HCC (38, 240, 241). Also, combination use of radiation and IL-12 could boost anti-tumor immunity by reducing MDSC accumulation and ROS production (242). Many potential targets of MDSCs have been designed to interfere with immature myeloid cells (**Table 3**), but their combination with anti-PD-1/PD-L1 blockades still require additional validation in preclinical and clinical models. Alternatively, inhibiting Tregs or TAMs is another strategy to restore immune response (258, 259). Treg can be depleted by numerous agents, such as cyclophosphamide, gemcitabine, mitoxantrone, fludarabine, and CCR4-targeted antibodies (253). Sorafenib, a multi-kinase inhibitor for HCC, is able to reduce Treg infiltration into the liver by downregulating the TGF- β signaling (54). It has been shown that WNT-β-catenin signaling induces M2-like polarization of TAM and thereby reinforces malignant behaviors, whereas blocking WNT-β -catenin pathway in TAMs may rescue immune evasion of HCC (260). Overall, the modulation of suppressive immune cells is a possible adjuvant therapy to attenuate HCC progression. As shown in **Table 3**, treatment of MDSCs, TAMs, and Tregs targets in HCC has been documented and could be a new strategy for treating HCC (254–257, 261).

**Table 3:** A summary of molecular targets in the tumor immune microenvironment of HCC.

Target cell	Molecule	Major effects	Therapeutic strategy	Reference
**MDSC**	CCL26	CCL26 mediates MDSC recruitment in the hypoxic regions of HCC.	CCL26 blockade	(39)
	CCL9/CCR1	CCL9/CCR1 induces MDSCs recruitment to the spleen.	CCL9/CCR1 blockade	(243)
	ENTPD2/CD39L1	HIF-1 prompts MDSC accumulation *via* ENTPD2/CD39L1 in HCC.	ENTPD2/CD39L1 blockade	(40)
	CCRK	CCRK induction drives mTORC1-dependent G-CSF expression to recruit MDSCs and enhance tumorigenicity in HCC.	Anti-CCRK	(244)
	IL-6	IL-6 expression level is highly associated with MDSC phenotype in HCC patients.	Anti-IL-6	(245)
	PD-L1	PD-L1^+^ MDSCs are increased in HCC patients.	PD-L1 blockade	(246)
	C5AR	C5AR can recruit MDSCs to the TIME.	C5AR blockade	(240)
**Treg**	PD-1	PD-1-mediated inhibitory signal in the TME.	PD-1 blockade	(247, 248)
	CTLA-4	Tumor-induced regulatory DC subset inhibit immunity *via* CTLA-4-dependent IL-10 and IDO production.	CTLA-4 blockade	(75, 249)
	TIM3	Antibodies against TIM3 restore immune response of HCC-derived T cells to tumor-specific antigens.	TIM3 blockade	(97, 250)
	LAG3	Antibodies against LAG3 restore immune response of HCC-derived T cells to tumor-specific antigens.	LAG3 blockade	(97)
	GITR	GITR-ligation can improve anti-tumor response by abrogating Treg-mediated suppression in HCC.	GITR blockade	(251)
	ICOS	ICOS^+^ FOXP3^+^ Treg cells are enriched in the HCC TME.	ICOS blockade	(252)
	CCR4	Tregs can be targeted and depleted by mABs towards CCR4.	Anti-CCR4	(253)
	TGF-β	TGF-β prompts Treg infiltration into the liver.	Sorafenib	(54)
**TAM**	IL-6, IL-23, IL-β, TNF-α	Cytokines enhance the expansion of IL-17-producing CD4^+^ Th17 cells.	Anti-IL-6, anti-IL-23, anti-IL-β, anti-TNF-α	(73, 254)
	TGF-β	TGF-β prompts TIM-3 expression in TAMs.	Anti-TGF-β	(66)
	IL-1β	IL-1β prompts EMT and HCC immune escape.	Anti-IL-1β	(68)
	CCR2	CCR2 prompts EMT transition and M2-plarization of TAMs.	Anti-CCR2	(255, 256)
	CSF-1	CSF-1 reprograms polarization of TAMs.	CSF-1 receptor antagonist	(257)

MDSC, myeloid-derived suppressor cell; Treg, regulatory T; TAM, tumor-associated macrophage; HCC, hepatocellular carcinoma; TME, tumor microenvironment; TIME, tumor immune microenvironment; CCL26, C-C motif ligand 26; ENTPD2, endothelial growth factor; IDO, indoleamine 2, 3-dioxygenase; HIF, hypoxia-inducible factor; G-CSF, granulocyte-colony-stimulating factor; CSF, colony-stimulating factor; DC, dendritic cell; mAB, monoclonal antibody; TNF-α, tumor necrosis factor α; TGF-β, transforming growth factor β; EMT, epithelial-mesenchymal transition.

TGF-β pathway is a promising target for HCC therapy, as its inhibition tends to reduce the EMT and reactivate NK cells. Galunisertib is a small molecular inhibitor that reduces the phosphorylation of SMAD2, downregulating TGF-β pathway and inhibiting HCC progression (262). Galunisertib monotherapy has been shown to extend overall survival of advanced HCC patients in a phase-II trial (263). Combination of galunisertib and sorafenib demonstrated an improvement of efficacy compared to historical records of sorafenib monotherapy (NCT01246986). The combination strategy of galunisertib and PD-1 blockade is ongoing in clinical trials (NCT02423343 and NCT02947165). The monoclonal anti-TGF-β antibody ascrinvacumab also showed hopeful results among HCC patients in a phase I-II trial (264) and its combinational application with nivolumab is currently under investigation (NCT05178043).

Targeting VEGF enables ICIs more effective through multiple pathways (265, 266). VEGF inhibition not only transiently normalizes abnormal vasculature, but also increases CTL infiltration and modulates checkpoint expression on T lymphocytes (267, 268). Therefore, VEGF inhibition appears to be an ideal combinatorial partner for ICI as a locoregional therapy for HCC. IMbrave150 trial demonstrated that the addition of anti-VEGF inhibitor (Bevacizumab) significantly improved efficacy from ICI (atezolizumab) (10). Other combinations of ramucirumab (anti-VEGFR2) or Lenvatinib (anti-VEGFR and anti-FGFR) with ICIs also have been investigated (269).

### 6.2 Harnessing the microbiome for HCC immunotherapy

Targeting the gut microbiota for HCC is increasingly attractive, including probiotics, prebiotics, fecal microbiota transplantation (FMT), and antibiotics. Since the gut microbiota dynamically regulates the host immunity, manipulating the gut microbiota may be a new orientation to improve anti-HCC immunotherapy.

Probiotics can keep gut microbial balance when given in certain amounts. Probiotic supplement as a dietary approach to repress HCC growth has been demonstrated. Feeding probiotics mixture Prohep (comprising *Lactobacillus rhamnosus* and *Escherichia coli*) could reduce liver tumor size, alter gut microbial composition to beneficial bacteria (*Oscillibacter* and *Prevotella*), and decrease the secretion of VEGF (270). Supplementing probiotics to Chinese subjects who are exposed to AFB1, such as *Lactobacillus rhamnosus* LC705 and *Propionibacterium*, could reduce the urinary excretion of aflatoxin-DNA adduct (AFB1-N7-guanine) (271). This finding kept in line with the protective capacity of probiotics against AFB1-induced HCC (272, 273). In another rat study, probiotics treatment containing *Lactobacilli*, *Bifidobacteria*, and *Streptococcus thermophilus subsp Salivarius*, can alleviate diethylnitrosamine (DEN)-induced hepatocarcinogenesis by preserving intestinal homeostasis and ameliorating chronic inflammation (154). Also, mice models treated with probiotics had a lower level of Th17 cells in gut compared to untreated mice. Therefore, probiotic can improve microecological balance, enhance intestinal barrier function, and prevent immune evasion of HCC.

Prebiotics are foods that selectively accelerate beneficial microorganism growth and suppress harmful bacterial growth, thereby adjusting gut microbial homeostasis (274). Besides, they can result in the production of short-chain fatty acid (SCFA) and ultimately inhibit HCC development. Prebiotics were found to maintain microbial stability and decrease pro-inflammatory pathways that trigger HCC initiation and progression (275). In mice given transplantation of BCR-ABL-transfected BaF3 cells, insulin-type fructans hold the promise to decrease hepatic BaF3 cell infiltration, relieve inflammation, and increase portal propionate content (276). Propionate inhibits BaF3 cell proliferation *via* cAMP-dependent pathway or by binding with GPR43 (276). Overall, prebiotics supplementation is a novel strategy to treat HCC.

Using antibiotics is another effective strategy to interrupt the tumor-prompting gut-liver axis. Antibiotics can reduce bacteria translocation, decrease pro-inflammatory signals from the leaky gut, and repress the synthesis of bacterial metabolites. For example, intestinal sterilization with antibiotic cocktail (containing neomycin, ampicillin, vancomycin, and metronidazole) has been proven to efficiently reduce the number and size of liver tumors induced by DEN-CCL4 or DMBA-HFD (179, 277). Consistently, the antibiotic cocktail (ABX, including vancomycin, primaxin, neomycin) or vancomycin treatment selectively elicited anti-tumor responses with increased CXCR6^+^ NK T cells and heightened IFN-*γ* production in HCC mouse models (178). As mentioned previously, CXCR6 expression level is controlled by gut microbiome-mediated primary-to-secondary bile acid conversion. A recent study suggests that vancomycin can inhibit HCC progression in insulin-fed TLR5-deficient mice (278). Concurrently, vancomycin can lead to selective depletion of gut microbiota, comprising *Bifidobacteria*, G^+^ *Lachnospiraceae*, and *Ruminococcaceae*.

FMT refers to the infusion of fecal solution from a healthy donor to the recipient intestinal tract to treat a disease associated with altered gut microbiota (279). FMT has successfully been used to treat *Clostridium difficile* infection *via* mechanisms including activation of mucosal immune system, maintenance of bile acid metabolism, and repair of the intestinal barrier (280). For example, alcohol-sensitive mice exhibited a decrease in *Bacteroidetes* and an increase in *Actinobacteria* following alcohol intake. After FMT, liver injury was relieved and dysregulated flora was partially recovered (281). Bajaj et al. reported that FMT enriched with *Lachnospiraceae* and *Ruminococcaceae* is able to restore the disruption of microbial diversity and function led by antibiotics in advanced cirrhosis patients (282). However, there are limited data on FMT in the treatment of HCC, and it is unclear whether microbial dysbiosis can be reverted by FMT (275). More studies are needed to validate the safety and efficacy of FMT in the future.

The significance of gut microbiota in modulating anti-tumor response to ICIs has been widely highlighted (283, 284). On the one hand, the dynamic change of gut microbiota can predict early outcome of immunotherapy. In a study, fecal samples from patients who respond to ICI showed higher taxa richness and more gene counts compared to non-responding patients (285). Stool fecal microbiota transplantation from cancer patients, who respond to ICIs, into germ-free or antibiotic-treated mice, ameliorated the efficacy of PD-1 ICIs, whereas fecal transplantation from non-responders failed to do so (286). This provoking finding is also supported by two other studies, describing different gut microbiota associated with improved response to ICIs (287, 288). Given those HCC patients with microbial dysbiosis, it is reasonable to speculate that the underlying dysbiosis potentially leads to immunotherapy failure. Microbial intervention may produce more profound effects in HCC than in other tumors. A feasible strategy is to combine ICI and selective microbiota manipulation. Recently, a clinical trial (NCT03785210) combining vancomycin treatment with ICI has been initiated, which will answer whether such a combination strategy would benefit patients with HCC. On the other hand, there is an association between the gut microbiota and immune-related toxicity (289). Targeting the specific microbiota may strengthen the effects of CTLA-4 blockade by reducing collateral toxicity (148).

### 6.3 Manipulating immunometabolism in the TME

The tumor-immune crosstalk inevitably leads to metabolic modifications in tumor cells and immune cells, serving as one of the most important mechanisms of immune evasion of HCC. Nutritional interventions aim to target immunometabolism in the TME (290). Dietary has been shown to have direct effects on both immune cells and tumor cells.

A ketogenic diet targets the Warburg effect in tumor cells by reducing glucose consumption while reprogramming effector T cells to rely on the OXPHOS (290, 291). In response to an increase of ketone bodies, CD4+ and CD8^+^ T cell secrete more cytokines, such as IFN-*γ*, TNF-α, perforin, and granzyme B (290). Nutritional interventions of essential amino acids also affect anti-tumor response. For example, arginine supplementation could switch T-cell metabolism from glycolysis to OXPHOS to enhance their survival (292, 293). Met supplementation might restore anti-tumor immunity by prompting the secretion of IL-2, TNF-α, and IFN-*γ* from TILs (294). IDO inhibition renders the TME less immunosuppressive by avoiding tryptophan depletion. It has been reported that IDO is involved in drug-resistance to ICI (295). Combinatorial treatments of IDO inhibitor and anti-PD1 or anti-CTLA4 blockades were shown to prolong survival in mouse models (295, 296). A phase I-II clinical trial (NCT03695250) is underway to evaluate IDO1 inhibitor (BMS-986205) in combination with nivolumab in patients with liver cancer. Caloric restriction is an alternative strategy to treat HCC. A study supported that caloric restriction in combination with radiation can decrease the abundance of Treg cells and expand the proliferation of CD8^+^ TILs in the TME (297). Moreover, it supports an immune signature linked to superior anti-tumor immunity and confers stem cell-like properties to effector T cells (298, 299). Altogether, targeting tumor-associated metabolic pathways is crucial to enhancing response to immune surveillance.

## 7 Conclusion

The tumor microenvironment of HCC is a dynamic and complicated network. Intricate interactions among suppressive immune cells, immunoregulatory cytokines or signaling, hostile metabolites, and the unbalanced gut microbiome collectively create a permissive TME that mediates immune evasion to favor HCC growth. In recent years, the combination therapy of atezolizumab and bevacizumab opened a new era for HCC treatment. However, HCC is still one of the worst prognoses and novel strategy targeting the TME is an urgent need. Given the complexity of the TME in HCC, combinatorial therapies can include ICIs, agents targeting immunosuppressive immune cells, anti-VEGF inhibitors, anti-TGF-β antibodies, microbiota manipulation, and metabolism intervention. A more holistic approach should be considered as a standard treatment for patients with advanced HCC. However, the molecular underpinnings governing immune evasion still need further clarification. Profound appreciation of the tumor-stromal interactions will enhance our understanding of the negative drivers of immunosurveillance. Multidimensional analysis, such as single cell analysis and next-generation sequencing technology, contribute to exploring detailed mechanisms behind HCC occurrence and identifying other targets in the TME.

## Author contributions

YQ designed the study and reviewed the manuscript. CC participated in study design and wrote the original draft of the manuscript. CC and ZW were mainly responsible for the design of tables and figures. YD contributed to the conception of the paper. All authors contributed to the article and approved the submitted version.
